# Spiking Neural Networks for Structural Health Monitoring

**DOI:** 10.3390/s22239245

**Published:** 2022-11-28

**Authors:** George Vathakkattil Joseph, Vikram Pakrashi

**Affiliations:** UCD Centre for Mechanics, Dynamical Systems and Risk Laboratory, School of Mechanical and Materials Engineering, University College Dublin, 4 Dublin, Ireland

**Keywords:** spiking neural network, neuromorphic, low-power, Loihi, cepstrum, Mahalanobis distance

## Abstract

This paper presents the first implementation of a spiking neural network (SNN) for the extraction of cepstral coefficients in structural health monitoring (SHM) applications and demonstrates the possibilities of neuromorphic computing in this field. In this regard, we show that spiking neural networks can be effectively used to extract cepstral coefficients as features of vibration signals of structures in their operational conditions. We demonstrate that the neural cepstral coefficients extracted by the network can be successfully used for anomaly detection. To address the power efficiency of sensor nodes, related to both processing and transmission, affecting the applicability of the proposed approach, we implement the algorithm on specialised neuromorphic hardware (Intel ® Loihi architecture) and benchmark the results using numerical and experimental data of degradation in the form of stiffness change of a single degree of freedom system excited by Gaussian white noise. The work is expected to open a new direction of SHM applications towards non-Von Neumann computing through a neuromorphic approach.

## 1. Introduction

Effective structural health monitoring (SHM) requires acquisition, transmission, and processing of multidimensional data over long periods of time. These typically comprise of acceleration, displacement, velocity, and strain data, along with environmental factors such as temperature [1,2]. To address the needs of acquiring high-resolution, high-volume data from a structure, distributed sensor networks are becoming popular [3]. Wireless sensor networks are favoured since they allow significant simplification of instrumentation in practice, and other benefits such as minimal invasion of host structure and remote reconfigurability [4]. Powering the sensor nodes, however, poses a challenge. Wiring for power from a grid partially offsets the advantages of wireless operation. Moreover, structures are often situated in remote or harsh areas with inadequate access to sources of electricity. Reliably powering such remote sensor nodes with appropriate processing capacity, along with information transmission, converts the problem to an energy management issue as well. This aspect consequently impacts all system design choices from sensor network topology, transmission rate, and on-node/off-node processing, amongst others [5].

Energy harvesters based on solar, thermal, wind, piezoelectric, or electromagnetic effects are being extensively studied to establish them as power source candidates for such scenarios [6]. The limited power available, however, from such devices is generally rationed for the acquisition and transmission phases, with the majority of the processing relegated to a central hub or off-site laboratory where access to sufficient processing power is available [5]. An optimisation problem of transmission is encountered here because Internet of Things (IoT)-focused networks such as LoRaWAN and Narrowband-IoT suffer from low bandwidth, whereas Wi-Fi suffers from short range [7,8,9]. Ideally, if damage-sensitive features (DSFs) [10] could be extracted from the data at the nodes themselves, the transmission required would be reduced to the feature vectors or their changes [11]. Methods and algorithms oriented towards this approach, as well as using the harvester itself as a sensor, have been reported [12,13,14,15,16]. However, currently, only basic processing can be achieved at the node with the energy generated by harvesters. Nevertheless, pattern recognition algorithms for SHM that adopt advances in machine learning have been proposed, with promising initial results [17,18,19,20,21]. The ability to implement such algorithms with limited power could prove to be a breakthrough in SHM technology.

A typical example of basic processing is transformation of a sensor input to the frequency domain. Even this transformation can be energy intensive, even with a fast Fourier transform (FFT) algorithm. However, the architecture used by the human ear for the qualitatively similar operation of spectral decomposition is extremely efficient. A linear scaling (*N*) in time and power is achieved [22] compared to an NlogN scaling for the FFT, where *N* is the number of output frequency bins. With this context, we consider neuromorphic computing, which is a relatively novel paradigm of hardware and software design implementing functional characteristics of such biological architectures on silicon [23,24]. The approach is especially suited to processes where a loss in precision is tolerable in exchange for greater energy efficiency and/or speed. SHM using distributed sensor nodes is a perfect candidate for a neuromorphic approach due to the demand for low-power computing of DSFs at the node level.

This paper shows the extraction of DSFs using a novel neuromorphic hardware platform and proposes its use in practice. The DSF vector is composed of coefficients generated using an approximate version of the power-cepstrum [25,26]. The cepstrum transformation has been long established as a method in condition monitoring of rotary machines [27,28]. The conceptual similarity of the transform to the processing in the human ear has led to its early adoption in audio-processing applications as well [29,30]. Application of cepstral analysis to SHM of civil infrastructure was proposed by a few authors recently [20,31,32,33,34,35].

It is observed that a neuromorphic approach can have a particular advantage in terms of computational and energy parsimony, while offering high pattern recognition capabilities [36,37,38]. A neuromorphic implementation of cepstral analysis for speech recognition and synthesis was shown recently [39]. As a first example of its kind, this paper demonstrates a neuromorphic implementation of cepstral coefficient extraction for SHM and implements it on the Intel^®^ Loihi neuromorphic platform [40]. The efficacy and power consumption is reported, showing promise for further investigation. While Loihi is still far from being a low-cost edge processor, SNN implementations in SHM, demonstration of solutions, and development of benchmarks of results create a pathway towards reaching this goal.

Recent proposals and simulations of utilising spiking neural networks for damage detection [41,42] showcase the potential of learning damage-sensitive features through backpropagation. However, a recurring challenge in the deployment of neural-network-based solutions in safety-critical applications is the explainability of the neural network model applied. In this work, an alternative approach is adopted where a minimal, yet well understood, feature extraction and anomaly detection method is converted to a spiking neural network. An experimental evaluation is performed on the neuromorphic platform Loihi to profile and validate the functionality. This demonstration could serve to lower the barrier to adoption of such technology for applications in SHM in different sectors [43,44].

## 2. Theory

Neurobiological systems are complex, and the field of neuroscience has made strides in understanding the functional characteristics of the core components that allow sensing and computation in the brain. This understanding has led to the advent of neuromimetic and neuromorphic devices where electronic components that capture these functional characteristics reproduce behaviour similar to the brain. To simplify the process of neuromorphic system design, multiple mathematical formalisms have been developed, allowing high levels of abstraction while preserving core aspects of behaviour. This work utilises the Neural Engineering Framework (NEF) formalism since it allows the construction of a spiking neural network of a **known transform** through an optimisation procedure as opposed to frameworks more suitable for learning a transformation based on input–output data. A brief summary of the formalism is outlined here.

### 2.1. Neural Engineering Framework

Encoding/decoding, transmission, and processing of information in the human brain is predominantly carried out through spiking activity of neurons. The neural engineering framework (NEF) provides a set of principles intended as a practical method for non-neuroscientists to transform traditional algorithms to a neuromorphic architecture using spiking (or nonspiking) neural networks [45]. The NEF allows the mapping of both linear and nonlinear dynamical systems to SNNs [46]. We use the NEF to build our network to extract the cepstrum. Spiking neural networks (SNNs) described here are distinct from the field of artificial neural networks, which has recently gained mainstream adoption [47]. There is no fitting or learning of weights from data involved in this application. The SNN in our case is fully defined based on the transformations to be performed (SNNs can be considered as a generalisation of conventional ANN and learning can be performed as well).

The three guiding principles of the NEF, pertaining to representation, transformation, and dynamics, are summarised below. A formal description of the principles is provided in Appendix A.

#### 2.1.1. Representation

**Figure 1:**
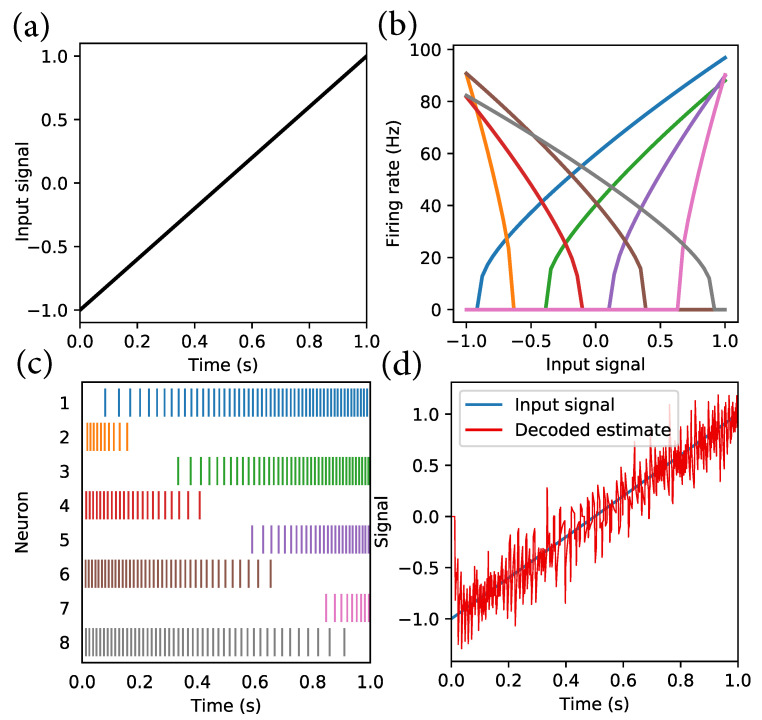
Illustration of encoding and decoding of signals using NEF. (**a**) normalised input signal, (**b**) tuning curves of 8 neurons, (**c**) spiking activity of neurons, (**d**) decoded signal compared with encoded signal [48].

A continuous time-varying signal of arbitrary dimensionality is to be represented by a population of spiking neurons. To achieve this, a nonlinear encoding and linear decoding scheme is used. Each neuron model is characterised by a tuning curve which defines its spiking activity as a function of its input current. Consider a signal, as shown in Figure 1a. The signal is to be encoded, for example, using eight neurons (representation error reduces with larger populations of neurons). The eight neurons are divided equally into those that spike due to positive signals and those that spike due to negative signals. The tuning curves of the eight neurons are shown in Figure 1b. The tuning curves have equally spaced intercepts in the range (−0.9,0.9). Based on the intercepts, the spiking activity starts and increases with signal amplitude in both cases. The spiking activity a(J) in each of the eight neurons representing the input signal is shown in Figure 1c. Note how the neurons with higher intercepts, such as neuron 7, start firing only at a high signal level. Thus, to represent a signal vector x, the corresponding current levels J driving the neurons can be determined as:

(1) $$J\left(x\right)=\alpha e\cdot x+J_{\mathrm{bias}}$$

where e are unit vectors (called encoders) that specify the direction of spiking (positive or negative) for each neuron, α is a scaling factor, and $J_{\mathrm{bias}}$ determines the intercept or the level at which spiking starts/stops (in Figure 1b, the lines are not not strictly straight since the neuron model is “leaky”. See Appendix A for more details).

Decoding of the spiking activity is performed using a weighted sum of the spiking activity of all neurons:

(2) $$\hat{x}=\sum_{i=0}^{n-1}d_{i}a_{i}$$

where $\hat{x}$ is the decoded estimate of x, $d_{i}$ is the set of decoding weight for each neuron, and $a_{i}$ is the spiking activity of the neuron. d is, in general, calculated by solving the least squares problem:

(3) Ad=X

where A is the activity of all neurons for all inputs and X is a sample input. With this representation scheme, any smooth signal can be encoded and decoded.

#### 2.1.2. Transformation

Arbitrary transformations of the signal can be decoded instead of the signal itself by simply solving for a different set of decoders d. For a sample signal *X*, Equation (3) becomes

(4) Ad=f(X)

where f(·) is the desired transform (when f(·) is known, decoders can thus be solved for. When only the output is known and the transformation is unknown, learning is involved).

#### 2.1.3. Dynamics

An arbitrary dynamical system can be simulated using a network of spiking neurons where the state vectors of the dynamical system are represented by ensembles of neurons. Recurrent connections of ensembles allow differential equations to be simulated. The work presented in this paper does not involve simulating the dynamics of a system and hence does not utilise the third principle.

### 2.2. Cepstrum

The cepstrum was proposed by Bogert et al. in 1963 [49] and later developed by Oppenheim and Schafer [25]. Interpretively, it can be considered as the “spectrum” of the logarithm of a spectrum. The (power) cepstrum is usually given by:

(5) $$C\left(x\left(t\right)\right)={\left|F^{-1}\left(\log\left({\left|F(x(t))\right|}^{2}\right)\right)\right|}^{2}$$

where F(x) denotes the discrete Fourier transform (DFT). In practice, the DFT is performed using the fast Fourier transform (FFT) algorithm, and the magnitude squaring at the end may be omitted. The echo delay or period of a periodic signal τ appears as a peak in the cepstrum reminiscent of frequency peaks in conventional Fourier analysis. The domain of the cepstrum is thus termed as the quefrency domain. The cepstrum is particularly sensitive to reverberations or echoes of a fundamental wavelet, whose form does not have to be known a priori. This allows it to be particularly useful in the analysis of seismic signals and human speech, where it was initially used, and, later, in condition monitoring of rotating machinery. Health monitoring of infrastructure can also benefit from the cepstrum as it can eliminate harmonics and deconvolve periodic/quasi-periodic signals from the waveform [20,31,32,33,34].

Since our objective is recognition, rather than reconstruction, of the signal, a compressed version of the cepstrum can be generated by substituting the inverse DFT with the inverse discrete cosine transform (DCT). DCT improves compression due to its reduced complexity, and comparisons exist in the classic literature [50]. Analogous to Fourier components, a set of cepstral coefficients can thus be extracted, capturing the most relevant information in the signal. Damage to the structure would manifest as a change in cepstral coefficients which can be analysed by averaging over time. Deviation from the baseline healthy condition can be evaluated using simple statistical measures such as the Mahalanobis distance. This approach to SHM has been proposed previously with variations of cepstral features, with good results [31,51].

The process of extracting the cepstrum, i.e., decomposition into the log-spectrum and compression into coefficients centred at a finite number of characteristic frequencies, has been shown to be very similar to the process of feature extraction in the human ear [39]. A variant of the cepstrum called Mel-frequency cepstrum, where the frequency bands are spaced based on the human auditory response, has been widely used in speech processing. Passive and active mechanical components in the ear, along with neurons, extract the feature vector from the incoming sound and encode it in spikes sent to the auditory cortex of the brain. Considering the energy efficiency of the process, which is of critical importance as described earlier, this paper looks at a neuromorphic approach to extracting cepstral coefficients analogous to Mel-frequency cepstral coefficients (MFCCs) but distinct in the extraction procedure. The approach was first described by Bekolay [39] for the purpose of speech recognition and synthesis. While lack of powerful SNN training algorithms has posed challenges in terms of their performance as compared to artificial neural networks, recent efforts [52,53] have established the potential advantages of SNN in the context of MFCC by retaining numerical efficiency along with low-energy aspects.

## 3. Methods

### 3.1. Overview

The objective is to create a neuromorphic implementation of the cepstrum and test the feasibility of using it for SHM. For this, simulated and experimental datasets of a one-degree of freedom (1-DoF) oscillator with a change in stiffness were used. Once validated theoretically, the algorithm was implemented on hardware. The “Loihi” chip developed by Intel^®^ was used to test the algorithm described in this paper. Loihi is a novel computing architecture composed of multiple cores composed of compartments of spiking neurons interconnected by synapses, allowing a direct mapping of a spiking neural network onto silicon [40]. The chip allows configuration of the network parameters such as synaptic delays as a hardware feature rather than emulated through algorithms. This allows SNNs to be flexibly implemented on the chip with minimal overhead, allowing high energy efficiency. The energy consumption per neuron update as well as synaptic operation (between neurons) is in the pico-Joule range on the chip, allowing it to achieve improvements in energy consumption of several orders of magnitude [54,55]. The SNN model was created using Nengo, a software library based on the NEF allowing an intuitive design environment [48], and deployed on the Loihi board using the Nengo Loihi backend.

### 3.2. Datasets

**Figure 2:**
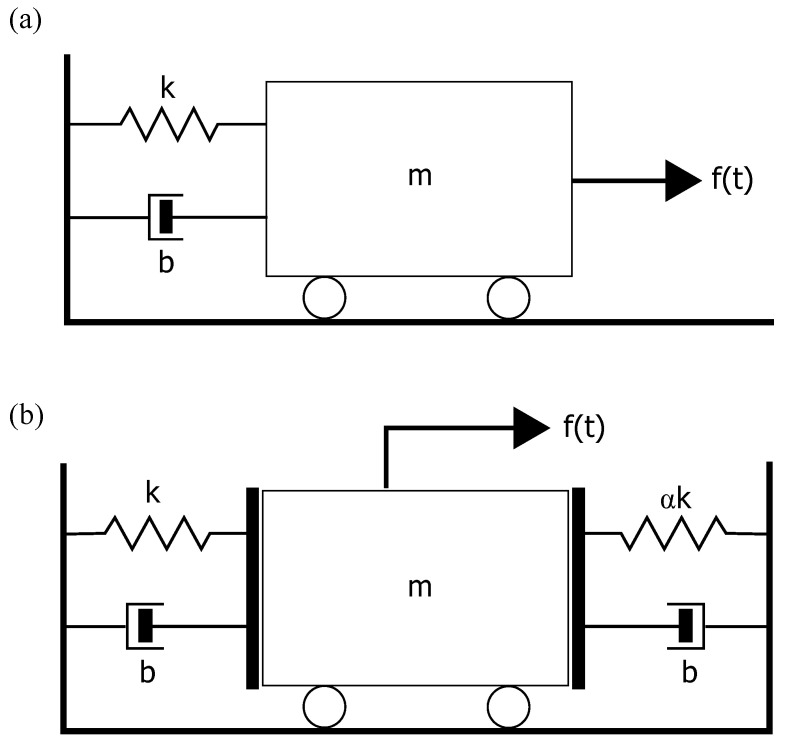
Schematic of simulated oscillator scenarios. (**a**) Linear and (**b**) bilinear 1-DoF systems were excited by a Gaussian white noise. Damage was introduced in the linear case by changing the stiffness k to 0.5 k. For the bilinear case, damage was introduced by changing α from 1 to 0.5.

The simulated dataset was produced by the excitation of a single-degree-of-freedom linear oscillator (Figure 2a) using Gaussian white noise forcing for 60 s. The acceleration of the oscillator was measured. After recording the time series of the baseline undamaged state, the model was excited by the same force vector but with a sudden stiffness change k${}^{\prime }$ = 0.5 k introduced at 30 s. The same procedure was then repeated for a bilinear oscillator (Figure 2b), where α is changed from 1 to 0.5 at 30 s.

**Figure 3:**
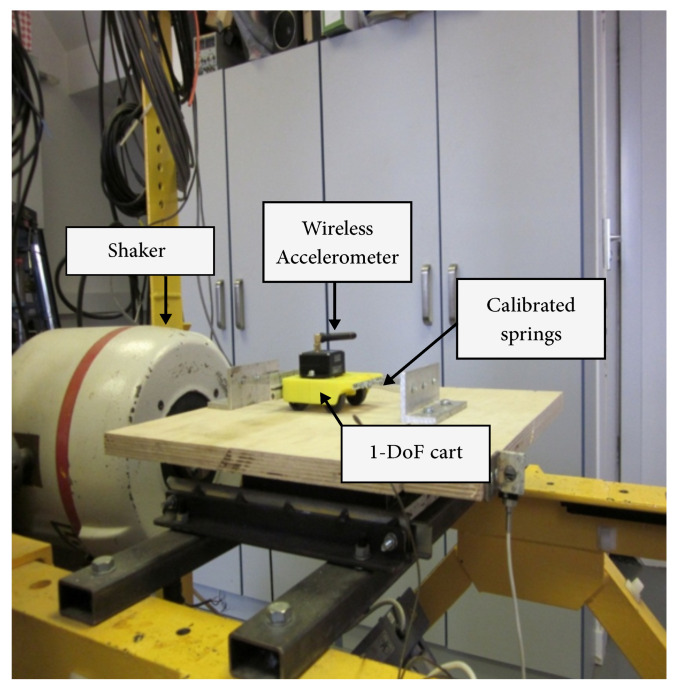
Setup for experimental test. The base on which the cart is placed was excited by Gaussian white noise by the shaker shown to the left. A total of 6 calibrated springs (3 on each side) are attached to the cart. The stiffness was changed in the damaged condition by removing 1 spring from each side.

The experimental dataset was produced by the experimental setup shown in Figure 3. A single-degree-of-freedom cart coupled to the frame by 6 springs was excited using white noise. To simulate damage, springs were removed from the cart. To simulate robustness against changes in surface morphology, the experiment was conducted on wood, sandpaper, and plastic surfaces. The acceleration was measured using an accelerometer attached to the cart sampled at a rate of 617 data points per second. A detailed description of the experiment can be found in [56].

### 3.3. Architecture

The cepstrum may be considered as the composition of three functions:

- F(·): Fourier transform;
- 2log(·): Log transform;
- $F^{-1}\left(\cdot \right)$: Inverse Fourier transform.

The final squaring operation may, in general, be omitted. The approach presented here is to use approximate methods to implement the transforms in a manner reminiscent of the human ear.

Passing the signals through a filterbank with different characteristic frequencies replicates the Fourier transform, as the output level of the filters provide an approximate frequency domain representation of the signal. Thus, a filterbank with logarithmically spaced characteristic frequencies would approximate the first two functions of the cepstrum, as listed above. For the third operation, the inverse discrete cosine transform (DCT-III or iDCT) may be used in place of the inverse Fourier transform as it generates an almost uncorrelated representation while compressing data dimensionality. More importantly, it can be very efficiently implemented on hardware compared to an FFT because it is limited to the real domain.

### 3.4. Filterbank

**Figure 4:**
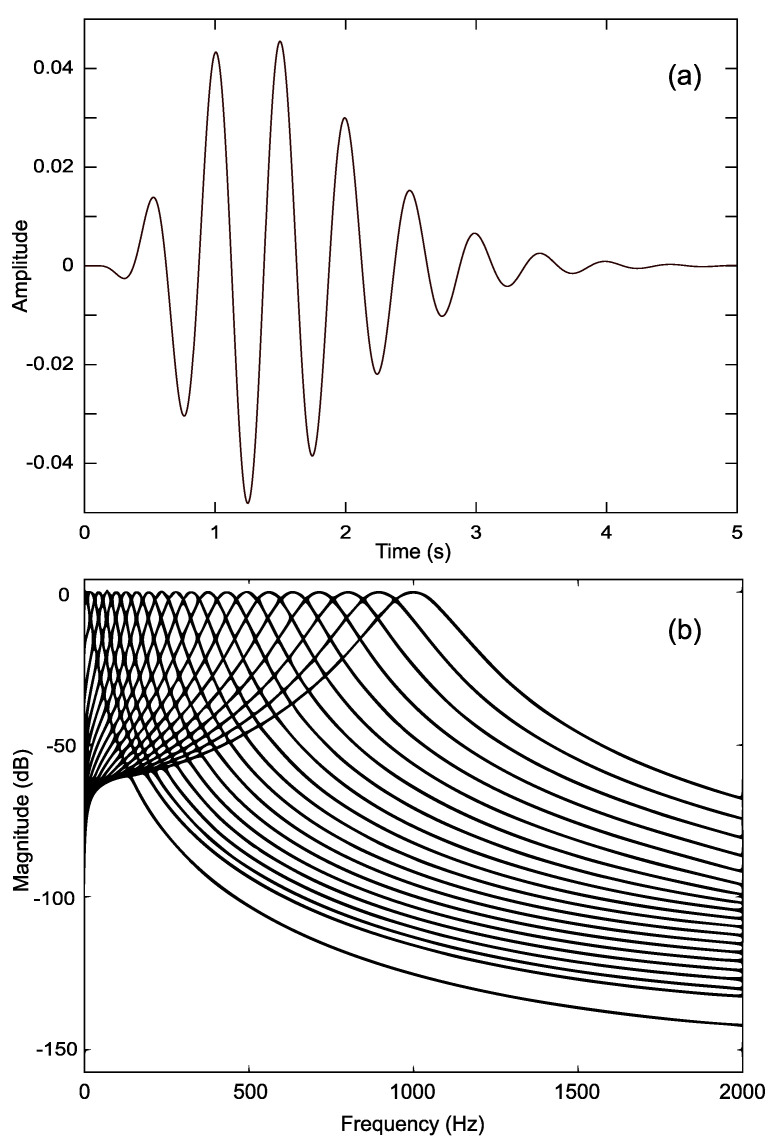
(**a**) Impulse response of gammatone filter. (**b**) Frequency response of filterbank (20/36 filters shown).

As indicated earlier, cepstral analysis of speech/audio signals often uses the Mel-frequency scale and triangular filters. The Mel-scale is an empirically derived range approximating the human auditory range from 20–20,000 Hz. For SHM, a more compact range suffices, as most structures have modal frequencies in the sub 2000 Hz range. Instead of triangular filters, the gammatone filter is used, which is among the most widely used auditory filters [57]. The gammatone filter can be implemented very efficiently and can process signals in near-real time [58]. The impulse response of the filter is given by:

(6) $$\begin{array}{cc} g(t) & =at^{n-1}e^{-2\pi bt\mathrm{ERB}(f)}\cos\left(2\pi ft\right),\; \end{array}$$

(7) $$\begin{array}{cc} \mathrm{ERB}(f) & =24.7+0.108f \end{array}$$

where *a* is the amplitude, *n* is filter order, *b* is the filter bandwidth parameter, and ERB(f) is the equivalent rectangular bandwidth of the filter centred at frequency *f*. A filterbank of 36 filters logarithmically spaced in the 0–2000 Hz range was used (Figure 4). Increasing the number of filters beyond this did not appear to affect the results perceivably.

### 3.5. SNN

**Figure 5:**
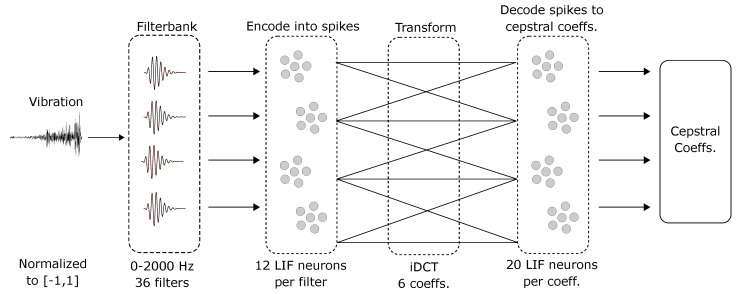
Schematic of the SNN architecture. The input signal is passed through filters with logarithmically spaced characteristic frequencies and encoded into spikes. An ensemble of spiking neurons is allocated to each filter. The inverse DCT transformation is performed on the output of the first layer and passed to the second layer. The transformation is performed through weights on the connections between the layers. The cepstral coefficients are decoded from the second layer. The number of coefficients to be extracted is chosen such that the L2-norm of all the coefficients combined captures >99% of the energy of the input signal. The number of ensembles is the same as the number of cepstral coefficients. The architecture is adapted from the Sermo model proposed by Bekolay [39].

The SNN involves 2 layers of neurons. Signals from the m-dimensional filterbank are encoded into spiking signals in the first layer (see Figure 5). Leaky-integrate-and-fire (LIF) type neurons are used to encode the information. Twelve neurons are used per characteristic frequency. Intercepts are uniformly distributed in the range (−0.1, 0.5) (the point along each neuron’s encoder where its activity starts). The connection between the first and second layer implements the inverse DCT. The second layer thus gives the cepstral coefficients which compose the feature vector. The dimensionality of the feature vector is decided using the energy contained in the cepstral coefficients estimated using the L2-norm. The number of coefficients from the iDCT in the feature vector is incremented by 1 until >99% of the energy of the original signal is contained in the vector. To represent each cepstral coefficient, an ensemble of 20 neurons was used.

The minimum number of cepstral coefficients required for the datasets in this study was found to be 6. Thus, for encoding the signal using 36 filterbanks, a total of 36 × 12 = 432 neurons were used. To decode the 6 iDCT coefficients, 6 × 20 = 120 neurons were used. The spiking neural network thus required 552 neurons to implement.

### 3.6. Damage Classification

The damaged state is identified using the Mahalanobis distance of the feature vector averaged in time from the distribution of the feature vector of the undamaged state. The Mahalanobis distance of vector x from the baseline multivariate distribution with mean vector μ is measured as

(8) $$D_{M}\left(x\right)=\sqrt{{\left(x-\mu \right)}^{T}S^{-1}\left(x-\mu \right)}$$

where *S* is the covariance matrix of the baseline distribution. An averaging window of 1.5 s is used. Threshold identification of the undamaged vs. damaged condition can be carried out based on tolerance levels required, as shown in Balsamo et al. [31].

## 4. Results

**Figure 6:**
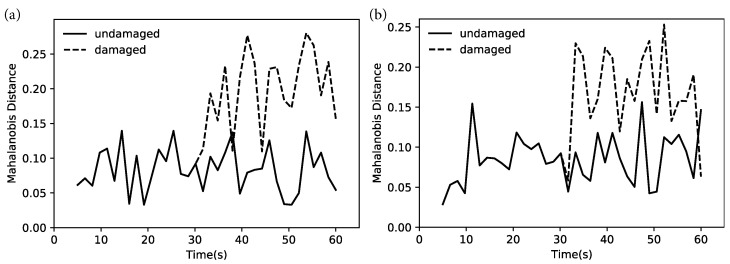
Change in Mahalanobis distance between the feature vectors of the damaged and undamaged states. (**a**) Simulated linear system and (**b**) simulated bilinear system. Damage introduced at t = 30 s.

The results for the simulated case (Figure 6) show a clear change in the cepstral coefficients after t=30 s, for both the linear and bilinear case, after which the Mahalanobis distance of the damaged state remains higher than the undamaged state overall. The oscillations in the Mahalanobis distance are smoothened if a higher averaging time (currently 1.5 s) is used, allowing a simple classification of damaged and undamaged states at very short timescales.

**Figure 7:**
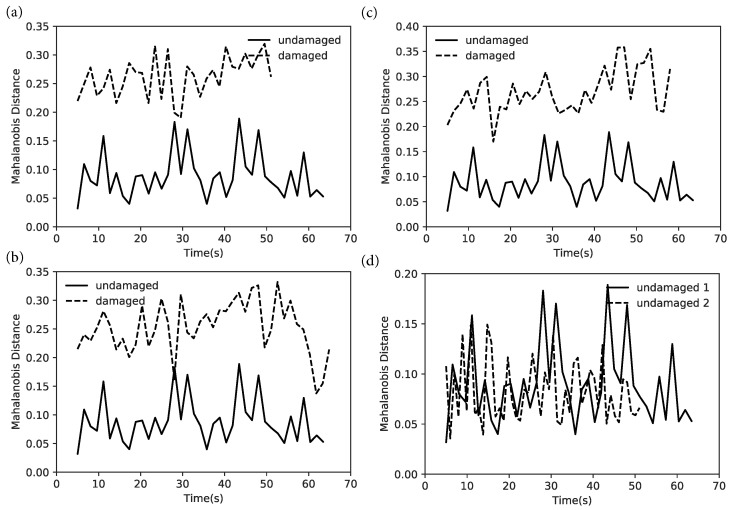
Change in Mahalanobis distance between the feature vectors of the damaged and undamaged states in experimental cases with surfaces: (**a**) Sandpaper, (**b**) plastic, and (**c**) wood. Mahalanobis distance comparison of the undamaged cases for wood and plastic is shown in (**d**).

For the experimental case, the baseline is the undamaged oscillation over a wood surface. Figure 7a–c show the Mahalanobis distances from the mean of the undamaged condition for sandpaper, plastic, and wood, respectively. All three plots show a clear differentiation in the feature vectors between damaged and undamaged, allowing a simple classification into damaged and undamaged states. The damaged states are always at a higher distance from the undamaged state. Figure 7d shows a comparison between undamaged conditions for two surfaces (wood and plastic), showing that the feature is robust to variations in surface conditions.

The static and dynamic power consumption of the chip were measured (using profiling tools provided through the Loihi SDK) to be approximately 1.0 Watt and 0.1 Watt, respectively.

## 5. Discussion and Conclusions

An SNN-based implementation is not expected to be more efficient than an optimised hardware implementation of the MFCC algorithm. However, the conceptual translation of a well-defined and well-understood transform to the spiking domain highlights the potential to use spiking neural networks as a programmable and explainable paradigm for feature extraction in SHM while also offering benefits of low-power operation. The scalability of neural networks to arbitrary complexity in feature extraction would also allow highly sophisticated pattern recognition from sensor streams across many degrees of freedom of the structure, or to enable sensor fusion from multimodal, multiresolution sensors.

Loihi contains 128 neuromorphic cores, each with 1024 spiking compartments. The implementation presented here utilises less than 1% of the available compartments on the chip. A fully utilised chip consumes less than 2 Watts, implying that multiple streams of sensor input could be processed in near-real time on a single chip to extract damage-sensitive features at a node. Alternatively, more advanced neuromorphic algorithms may be implemented at the node level to extract better damage-sensitive features to be transmitted.

Static power dominates the power consumption in Loihi at ∼1 W. However, an application-specific integrated circuit (ASIC) for SHM could be envisaged with a subset of neurons and optimised functions for pre-/post-processing that brings the resource utilisation closer to 100% operating at the mW range or lower. Several digital and mixed-signal implementations of spiking neural network accelerators have been developed recently that consume power as low as 100 μW [59,60,61,62,63]. The prospects that these architectures offer are perfectly suited for SHM since true edge deployment of sophisticated damage-detection algorithms may become viable once a sufficiently low threshold of system power (including acquisition and transmission) is crossed.

The damage identification using Mahalanobis distance was carried out using feature vectors from a single sensor. An averaging window of just 1.5 s results in a clear distinction in the damaged and undamaged state, as shown. This confirms that the SNN-based cepstrum generates damage-sensitive features. It is reasonable to project that integration of data from multiple sensors over larger windows of time along with pattern recognition algorithms suited for neuromorphic implementation would allow accurate damage identification at very low power levels.

The novelty introduced in this work is the use of a neuromorphic architecture (non-Von Neumann) as opposed to a conventional processing architecture (such as x86). The cepstrum, by virtue of its biological similarity, lends itself to a simple adaptation. However, arbitrary transformations of the input signal can be implemented using the methods as described in the NEF. Algorithms that rely on incorporate online learning rather than a predetermined explicit transform would gain higher advantages from an SNN implementation. The prospects offered by such advanced processing capabilities at a node with low power demand open avenues for SHM implementation that were not feasible previously.

## Data Availability

All data are available upon reasonable request.

## Appendix A

The Neural Engineering Framework (NEF) allows arbitrary dynamical systems to be described using spiking or nonspiking neural networks. A formal description of the framework is presented below. The NEF can approximate exact mathematical operations with precision of $O(\sqrt{n})$ for spiking networks and O(n) for the nonspiking case. The framework can be summarised using three principles: representation, transformation, and dynamics.

**Principle 1: Representation**

The scheme of distributed information representation in the NEF is called population coding, where an ensemble of neurons collectively encode information. The scheme was proposed in 1986 based on empirical evidence from the monkey cortex. A spiking neuron is a biologically derived model where the neuron “fires” only when the membrane potential, an intrinsic property of the neuron, crosses a threshold. It is also called the integrate-and-fire model. We represent the model using a capacitance *C*:

(A1) $$\frac{dV(t)}{dt}=\frac{1}{C}J\left(t\right)$$

where the voltage V(t) increases with time until it reaches a threshold, when a spike is emitted and the voltage is reset. A biological implausibility in this model is that it retains any energy input indefinitely. To overcome this, a leaky integrate-and-fire (LIF) neuron model was proposed, which can be represented as:

(A2) $$\frac{dV(t)}{dt}=\frac{1}{RC}\left(J(t)R-V(t)\right)$$

Incorporating a refractory period after each spike $\tau_{\mathrm{ref}}$, during which spiking cannot happen for a constant input (thereby limiting spike frequency), the activity of a neuron can be solved exactly as

(A3) $$s\left(J\right)=\left\{\begin{array}{cc} \frac{1}{\tau_{\mathrm{ref}}-\tau_{\mathrm{RC}}\ln\left(1-\frac{J_{\mathrm{th}}}{J}\right)} & \;\mathrm{if}\;J>J_{\mathrm{th}}\\ 0 & \;\mathrm{Otherwise} \end{array}\right.$$

where *J* is the input current, $J_{\mathrm{th}}$ is the threshold at which spiking occurs, and $\tau_{\mathrm{RC}}$ is the time constant for the rate of charge accumulation.

Using the LIF neuron model, the NEF describes a nonlinear encoding and linear decoding scheme to represent a *q*-dimensional vector in a population of *n* spiking neurons. Each neuron’s activity *a* is a function of the current *J* injected into it, e.g., spiking begins in a neuron at a current threshold and the spike rate increases with current. A different neuron may have the inverse behaviour, where spiking decreases with current and stops at a current threshold. An ensemble of *n*-neurons of both “positive” and “negative” spiking directions as n→∞ can thus exactly represent arbitrary signals using thresholds at all real numbers. The input current vector to encode the signal vector *x* is thus:

(A4) $$J\left(x\right)=\alpha e\cdot x+J_{\mathrm{bias}}$$

where $J_{\mathrm{bias}}$ determines the $J_{\mathrm{th}}$ (intercept/threshold) of each neuron, e gives the direction, and α gives the rate of increase of the spiking frequency with signal amplitude. In practice, a finite domain of [−1, 1] is used, scaling the signals to the domain, and outliers are effectively clipped at the boundaries. Intercepts are typically distributed equally between positive and negative directions sampled uniformly (see Figure 1). However, to optimise the usage of each neuron, the choice of intercepts may be skewed or shifted based on the system modelled, e.g., if only the threshold-crossing events of a signal in the positive direction, say ≥0.9, are to be encoded, all encoders e may be set to be positive and all elements of $J_{\mathrm{bias}}$ may be set to 0.9. The activity of the ensemble using Equation (A3) is thus a(J), where the transfer function of a single neuron, as shown in Equation (A3), is used, giving

(A5) a(J)=s(J)

Decoding encoded signals from the activity a(J) amounts to estimating the vector x as the weighted sum of each neuron’s activity. The weights are called decoders in the NEF.

(A6) $$\hat{x}=\sum_{i=1}^{n}d_{i}a_{i}$$

where $\hat{x}$ is the decoded estimate of x, $d_{i}$ is the set of decoding weights for each neuron, and $a_{i}$ is the spiking activity of the neuron. Since the characteristics of each neuron are known, the activity generated by all possible inputs is also known. This allows the decoding weights to be solved for by setting up the equation

(A7) Ad=X

where X is set of sample inputs and A is the set of activities of all neurons *n* for all possible inputs *m*, given by

(A8) $$A=a_{j}\left(X_{i}\right)$$

where i=1,2,…,m and j=1,2,…,n. An approximation of the decoders d is obtained by solving the least squares problem given by Equation (A7).

Since the spikes generated by the neurons take a finite amount of time to decay, a post-synaptic filter h(t) is introduced to the decoding operation. A basic model for the filter is as a decaying exponential:

(A9) $$h\left(t\right)=\frac{1}{\tau_{\mathrm{PSC}}}e^{-t/\tau_{\mathrm{PSC}}}$$

Thus, the activity of a neuron is

(A10) $$a\left(t\right)=\sum_{s}\delta \left(t-t_{s}\right)*h\left(t\right)=\sum_{s}h\left(t-t_{s}\right)$$

where δ is the Dirac delta function for the spikes, *s* is the set of spike times, and * represents convolution.

The decoded estimate $\hat{x}$ is then:

(A11) $$\hat{x}=\sum_{i=1}^{n}\sum_{s}h\left(t-t_{i,s}\right)d_{i}$$

**Principle 2: Transformation**

The connections between neurons allow transformations of the encoded signal before decoding using weights on the connections. The activity of *n* neurons from an ensemble acts as the input current to another neuron *j* in an ensemble:

(A12) $$J_{j}\left(x\right)=\sum_{i=1}^{n}\omega_{ij}a_{i}+J_{j,\mathrm{bias}}$$

where $\omega_{ij}$ is the connection strength between neurons *i* and *j*. If no transformation is applied on the signal, using Equations (A4) and (A6), the weights can be expressed as

(A13) $$\omega_{ij}=\alpha_{j}e_{j}\cdot d_{i}$$

If a linear transformation is to be implemented on the signal, the input current to the downstream ensemble can be modified by changing $\omega_{ij}$ as

(A14) $$\omega_{ij}=\alpha_{j}e_{j}L_{\mathrm{ji}}d_{i}$$

where $L_{ji}$ gives the transformation matrix. Thus, for linear transformation, the decoders are simply scaled using the transformation matrix to obtain the transformed output from the network.

For an arbitrary function f(x), the computation of weights, and consequently decoders, becomes an optimisation problem, as shown below.

The estimate of the output to be obtained after decoding is

(A15) $$\begin{array}{cc} \left(f(x)*h\right)\left(t\right) & \approx \sum_{i=}^{n}\left(a_{i}*h\right)\left(t\right)d_{i} \end{array}$$

(A16) $$\begin{array}{cc} \; & =\sum_{i=1}^{n}\sum_{s}h\left(t-t_{i,s}\right)d_{i} \end{array}$$

The activity of a single neuron for constant input is given by Equation (A3). Introducing a white noise process η to account for the variance of spiking across the population, the solution to the set of decoders *d* in Equation (A16) is given by the minimisation problem:

(A17) $$d=\underset{R^{n\times q}}{\mathrm{argmin}}\int_{m}{∥f\left(v\right)-\sum_{i=1}^{n}\left(s_{i}\left(v+\eta \right)\right)d_{i}∥}_{2}^{2}dv$$

where *m* is the domain of the *q*-dimensional signal x and v∈m. The optimisation does not depend on the nature of *x*, but only on the distribution of the signal. The dynamic problem involving spiking has been converted to a static problem, allowing the decoders to be computed without explicitly simulating the neurons in time. This convex optimisation problem can be solved by sampling the domain *m* uniformly and solving the resulting least-squares problem, similar to Equation (A7).

**Principle 3: Dynamics**

A linear time-invariant (LTI) system can be represented as

(A18) $$\begin{array}{cc} \dot{x}\left(t\right) & =Ax(t)+Bu(t) \end{array}$$

(A19) $$\begin{array}{cc} y(t) & =Cx(t)+Du(t) \end{array}$$

The ability to simulate dynamical systems relies on the availability of an integrator, i.e., the source of dynamics is the integration over time of the system above. In the NEF, the integrator is replaced by a *leaky integrator* given by the low-pass filter h(t) available on the connections between the neurons.

(A20) $$h\left(t\right)=\frac{1}{\tau }e^{-t/\tau }=L^{-1}\left(\frac{1}{1+\tau s}\right)$$

where $L^{-1}\left(\cdot \right)$ is the inverse Laplace transform.

Thus, a recurrent connection in an ensemble allows the integration of Equation (A19) by replacing *A* and *B* as follows:

(A21) $$\begin{array}{cc} A^{\prime } & =\tau A+I \end{array}$$

(A22) $$\begin{array}{cc} B^{\prime } & =\tau B \end{array}$$

where I is the identity matrix. In other words, the replacement of the integrator by the filter is compensated by driving the synapse with $\tau \dot{x}\left(t\right)+x\left(t\right)$. This transformation of x(t) may be implemented using Principle 2, thereby allowing simulation of dynamical systems.
